# δ‐cells and β‐cells are electrically coupled and regulate α‐cell activity via somatostatin

**DOI:** 10.1113/JP274581

**Published:** 2017-11-02

**Authors:** L. J. B. Briant, T. M. Reinbothe, I. Spiliotis, C. Miranda, B. Rodriguez, P. Rorsman

**Affiliations:** ^1^ Oxford Centre for Diabetes, Endocrinology and Metabolism, Radcliffe Department of Medicine University of Oxford Oxford OX3 7LE UK; ^2^ Department of Computer Science University of Oxford Oxford OX1 3QD UK; ^3^ Metabolic Physiology, Department of Physiology, Institute of Neuroscience and Physiology University of Gothenburg SE‐405 30 Gothenburg Sweden

**Keywords:** alpha cell, delta cell, beta cell, Islet cell, computer modelling, electrophysiology, somatostatin, optogenetics

## Abstract

**Key points:**

- We used a mouse expressing a light‐sensitive ion channel in β‐cells to understand how α‐cell activity is regulated by β‐cells.
- Light activation of β‐cells triggered a suppression of α‐cell activity via gap junction‐dependent activation of δ‐cells.
- Mathematical modelling of human islets suggests that 23% of the inhibitory effect of glucose on glucagon secretion is mediated by β‐cells via gap junction‐dependent activation of δ‐cells/somatostatin secretion.

**Abstract:**

Glucagon, the body's principal hyperglycaemic hormone, is released from α‐cells of the pancreatic islet. Secretion of this hormone is dysregulated in type 2 diabetes mellitus but the mechanisms controlling secretion are not well understood. Regulation of glucagon secretion by factors secreted by neighbouring β‐ and δ‐cells (paracrine regulation) have been proposed to be important. In this study, we explored the importance of paracrine regulation by using an optogenetic strategy. Specific light‐induced activation of β‐cells in mouse islets expressing the light‐gated channelrhodopsin‐2 resulted in stimulation of electrical activity in δ‐cells but suppression of α‐cell activity. Activation of the δ‐cells was rapid and sensitive to the gap junction inhibitor carbenoxolone, whereas the effect on electrical activity in α‐cells was blocked by CYN 154806, an antagonist of the somatostatin‐2 receptor. These observations indicate that optogenetic activation of the β‐cells propagates to the δ‐cells via gap junctions, and the consequential stimulation of somatostatin secretion inhibits α‐cell electrical activity by a paracrine mechanism. To explore whether this pathway is important for regulating α‐cell activity and glucagon secretion in human islets, we constructed computational models of human islets. These models had detailed architectures based on human islets and consisted of a collection of >500 α‐, β‐ and δ‐cells. Simulations of these models revealed that this gap junctional/paracrine mechanism accounts for up to 23% of the suppression of glucagon secretion by high glucose.

## Key points

- We used a mouse expressing a light‐sensitive ion channel in β‐cells to understand how α‐cell activity is regulated by β‐cells.
- Light activation of β‐cells triggered a suppression of α‐cell activity via gap junction‐dependent activation of δ‐cells.
- Mathematical modelling of human islets suggests that 23% of the inhibitory effect of glucose on glucagon secretion is mediated by β‐cells via gap junction‐dependent activation of δ‐cells/somatostatin secretion.

**Abbreviations**
CARB: carbenoxolone
ChR2: channelrhodosin‐2 (H134R)
Cx36: connexin‐36
GIRK: G‐protein inwardly rectifying potassium
GJ: gap junction
RFP: red fluorescent protein
RIP: rat insulin promoter
SST: somatostatin
SSTR2: somatostatin receptor type 2
T2DM: type 2 diabetes mellitus
YFP: yellow fluorescent protein

## Introduction

Type 2 diabetes mellitus (T2DM) is a metabolic disorder typically characterized by hyperglycaemia, insulin resistance and insufficient insulin secretion from islet β‐cells (Leahy, 2005; American Diabetes Association, 2010). However, it is becoming increasingly apparent that over‐secretion of glucagon from islet α‐cells also contributes to the increased hepatic glucose production and associated hyperglycaemia in T2DM. The abnormalities in glucagon secretion in T2DM include both loss of adequate suppression under hyperglycaemic conditions and insufficient release during episodes of hypoglycaemia (Cryer, 2002, 2008; Cryer *et al*. 2003; Dunning *et al*. 2005; D'Alessio, 2011; Unger & Cherrington, 2012). This has driven efforts to understand the mechanisms regulating α‐cells and glucagon secretion.

The glucagon‐producing α‐cells reside in a ‘paracrine environment’; islets are multicellular micro‐organs that comprise predominantly α‐, β‐ and δ‐cells (Kim *et al*. 2009). Regulation of glucagon secretion involves both intrinsic and paracrine mechanisms. The significance of paracrine regulation of α‐cells is one of the most contested aspects of islet cell biology (Gromada *et al*. 2007; Jacobson *et al*. 2009; Walker *et al*. 2011; Gylfe, 2013, 2016; Gylfe & Gilon, 2014; Briant *et al*. 2016). Numerous paracrine factors have been demonstrated to be important for regulating glucagon secretion from α‐cells, including insulin (Franklin *et al*. 2005; Ravier & Rutter, 2005), serotonin (Almaca *et al*. 2016), somatostatin (Hauge‐Evans *et al*. 2009) and urocortin3 (van der Meulen *et al*. 2015). Understanding how paracrine factors regulate glucagon secretion is fundamental to our understanding of the pathophysiology of T2DM, because the paracrine environment is known to be altered in this disease (Rahier *et al*. 1983; Kilimnik *et al*. 2011).

Optogenetic strategies have been successfully used in neurophysiology to study cell‐to‐cell communication for over a decade (Deisseroth, 2015; Cerritelli *et al*. 2016). Recently, this technique has also been used to study β‐cell physiology (Reinbothe *et al*. 2014; Johnston *et al*. 2016), but has not yet been used to elucidate the paracrine regulation of α‐cells. This strategy is the perfect experimental paradigm for studying the paracrine regulation of α‐cell activity because, in contrast to pharmacological approaches, it affords precise temporal and spatial control of paracrine signals from the other cell types.

In this study, we employed this strategy by using islets from mice expressing channelrhodopsin‐2 (ChR2) specifically in β‐cells in a transgenic cross of a floxed‐ChR2 line and a mouse line expressing Cre under the rat insulin promoter (RIP; Reinbothe *et al*. 2014). As these channels are light‐gated, β‐cells from these mice can be electrically activated by exposing whole islets or even single cells to 488 nm light (‘opto‐activation’). Our mouse model allowed control of electrical activity in β‐cells, which we could modulate whilst recording from α‐ and δ‐cells using patch‐clamp electrophysiology and Ca^2+^ imaging. Here we provide evidence for a regulatory network extending from the β‐cells, via the δ‐cells, to the α‐cells that involves both electrical transmission (via the gap junctions) and diffusion of secreted (paracrine) factors. We also evaluated the functional significance of these mechanisms by constructing and simulating computational models of human pancreatic islets.

## Methods

### Ethical approval

All animal experiments were conducted in accordance with the UK Animals Scientific Procedures Act (1986) and University of Oxford and Gothenburg University ethical guidelines, and were approved by the respective local Ethics Committees.

### Animals used in this study

Mice expressing ChR2 (H134R) and yellow fluorescent protein (YFP) under RIP (RIPCre^+/−^ChR2‐YFP^+/−^ mice) were generated as previously described (Reinbothe *et al*. 2014). Electrical activity in β‐cells in these mice can be triggered by exposing the islet to 488 nm light. To facilitate identification of δ‐cells in intact islets we also used islets from mice expressing a red fluorescent protein (RFP) reporter in δ‐cells under the somatostatin (SST) promoter (SST‐RFP; Egerod *et al*. (2015)).

### Preparation of pancreatic islets

Mice of both sexes (age = 120 ± 10 days) were killed by cervical dislocation (Schedule 1 procedure). Pancreatic islets were isolated by liberase digestion. Islets were used acutely and were, pending the experiments, maintained in tissue culture for <24 h in RPMI medium containing 7.5 mm glucose prior to the measurements.

### Calcium imaging in islet cells in response to optogenetic stimulation of β‐cells

Islets from RIPCre^+/−^ChR2‐YFP^+/−^ mice were incubated on Cell‐Tak‐coated glass‐bottom dishes (BD Biosciences, Franklin Lakes, NJ, USA) overnight in RPMI and 10 mm glucose (10G). The islets were washed with bath solution (containing 2.8 mm glucose; 2.8G) and loaded with the calcium dye Rhod‐2 (5 μm; Thermo Fisher Scientific, Inc., Waltham, MA, USA) for 45 min at 32°C, washed and again incubated in bath solution for 30 min at 32°C. Bath solution consisted of (in mm) 140 NaCl, 3.6 KCl, 0.5 MgSO_4_·7H_2_O, 1.5 CaCl_2_ and 10 Hepes (pH 7.4 with NaOH). The islets were then continuously perfused (1 ml min^−1^) with 2.8 mm glucose at 32–33°C on a Zeiss LSM 700 confocal microscope. The membrane of a single YFP^+^ β‐cell was then photo‐stimulated using the bleaching function of the 488 nm laser line with 25 μs pixel dwell time. This was executed at 70% power (7 mW fibre output) with 10 frame intervals between each stimulus. The intracellular Ca^2+^ ([Ca^2+^]_i_) response to this optogenetic stimulation could then be observed in YFP^−^ (non‐β) cells of the same islet loaded with Rhod‐2 (555 nm channel). Cells that were spontaneously active at 2.8G (likely to be α‐cells; Le Marchand & Piston, 2010) were not used for these experiments, which were intended to focus on YFP^−^ cells inactive at 2.8G (likely to be δ‐cells). Recent studies have demonstrated that some δ‐cells are active in low glucose (Shuai *et al*. 2016), suggesting that our selection criteria may erroneously discard some δ‐cells. However, the cells selected for analysis comprised 10 ± 4% of the cells in the confocal section (*n* = 8 islets), in fair agreement with the fraction of δ‐cells in mouse islets (Cabrera *et al*. 2006). These considerations argue that our measurements principally reflect the behaviour of δ‐cells. Regions of interest were restricted to the centre of the cell, avoiding the membrane, to minimize the risk of contamination of the quantified fluorescence by neighbouring cells.

### Patch‐clamp electrophysiology

Islets isolated from RIPCre^+/−^ChR2‐YFP^+/−^, non‐ChR2‐expressing littermate controls and SST‐RFP mice were also used for patch‐clamp electrophysiological recordings. These recordings (in intact islets) were performed at 33–34°C using an EPC‐10 patch‐clamp amplifier and PatchMaster software (HEKA Electronics, Lambrecht/Pfalz, Germany). Currents were filtered at 2.9 kHz and digitized at >10 kHz. A new islet was used for each recording.

#### Standard whole‐cell recording of GJ currents

Gap junction (GJ) currents were recorded from RFP^+^ δ‐cells in SST‐RFP mice, under the standard whole‐cell configuration. Only recordings with an access resistance of <50 MΩ were used for analysis. The pipette solution consisted of (in mm) 120 KCl, 1 CaCl_2_, 1 MgCl_2_, 10 Hepes (pH 7.15 with KOH), 3 MgATP and 0.05 EGTA. The bath solution consisted of (mm) 138 NaCl, 5.6 KCl, 5 Hepes, 1.2 MgCl_2_·6H_2_O and 2.6 CaCl_2_ (pH 7.4 with NaOH).

GJ currents evoked in 20 mm glucose were recorded by clamping the membrane at −70 mV and continuously recording the membrane current. The GJ inhibitor carbenoxolone (CARB; Sigma‐Aldrich, St Louis, MO, USA; Juszczak and Swiergiel (2009)) was used at a concentration of 100 μm to block GJ currents. GJ current amplitude was analysed in the presence of 20 mm glucose before, during and after addition of CARB. RFP^+^ cells were also identified as δ‐cells by ‘electrophysiological fingerprinting’, computed by using a multinomial logistic regression function which can identify islet cell type with 94% accuracy (Briant *et al*. 2017).

#### Membrane potential recordings in islet cells in response to optogenetic stimulation of β‐cells

Membrane potential recordings were conducted on RIPCre^+/−^ChR2‐YFP^+/−^ and non‐ChR2‐expressing littermate controls using the perforated patch‐clamp technique, as previously described (De Marinis *et al*. 2010). The pipette solution consisted of (mm) 76 K_2_SO_4_, 10 NaCl, 10 KCl, 1 MgCl_2_·6H_2_O and 5 Hepes (pH 7.35 with KOH). For these experiments, the bath solution contained (mm) 140 NaCl, 3.6 KCl, 10 Hepes, 0.5 MgCl_2_·6H_2_O, 0.5 Na_2_H_2_PO_4,_ 5 NaHCO_3_ and 1.5 CaCl_2_ (pH 7.4 with NaOH). Amphotericin B (40 mg ml^−1^, Sigma) was added to the pipette solution to give electrical access to the cells (series resistance of <100 MΩ). α‐cells and δ‐cells were confirmed by absence of YFP, glucose‐induced electrical activity and the aforementioned logistic regression model (Briant *et al*. 2017). The recording chamber was then shielded from ambient light and ChR2‐expressing cells were stimulated with 488 nm light pulses (10 ms duration, 20 Hz) using a fibre‐coupled LED with a collimator (WT&T, Pierrefonds, QC, Canada), triggered by the HEKA amplifier. Similar experiments were conducted in littermate controls to ensure electrical responses were not due to the Becquerel effect. The power output of the light source was calibrated to deliver 0.5 mW mm^−2^ to the recording chamber.

All perforated patch‐clamp recordings with opto‐activation were conducted at 5 mm glucose. Preliminary experiments demonstrated that opto‐activation of β‐cells at low glucose concentration produced small (<15 mV) amplitude spikes (due to the low input resistance). At 5 mm glucose, K_ATP_ channels in β‐cells are known to be mostly shut (Trube *et al*. 1986), which would facilitate opto‐activation of these cells. We therefore chose 5 mm glucose as the experimental condition because we wanted to be close to the threshold for β‐cell firing (EC_50_ = 8.3 mm glucose; Antunes *et al*. 2000), but also sub‐threshold to that producing maximal inhibition of glucagon secretion (6 mm glucose; Walker *et al*. (2011)).

### Hormone secretion measurement

Islets from RIPCre^+/−^ChR2‐YFP^+/−^ mice were incubated overnight in RPMI (7.5 mm glucose; 7.5G) in a cell culture incubator. Size‐matched batches of 20 islets were pre‐incubated in 0.3 ml of modified Krebs–Ringer buffer with 2 mg ml^−1^ bovine serum albumin (KRB) and 3G, for 1 h in a water bath at 37⁰C. This was followed by a 1 h incubation in 0.2 ml KRB supplemented with 5 mm glucose in the dark. The medium was removed and transferred to dry ice, and the islets were further incubated in 0.2 ml KRB with 5 mm glucose in the presence of 488 nm light pulses (20 Hz, 10 ms duration) for 1 h. The medium was again removed and transferred to dry ice. For hormone content measurements, the islets were lysed in 0.1 ml of acidic ethanol, followed by sonication on ice for 10 s. Insulin measurements were performed using a mouse insulin assay system (Meso Scale Discovery, Rockville, MD, USA), glucagon measurements were performed using the Millipore RIA system and SST measurements were performed using the Somatostatin EURIA radioimmunoassay (Eurodiagnostica, Malmö, Sweden), which is specific for somatostatin‐14 (the somatostatin secreted by the pancreatic δ‐cells).

### Statistical tests and time‐series analysis of experimental data

All data are reported as mean ± SEM, unless otherwise stated; ‘*n*’ refers to the number of cell recordings and ‘*N*’ to the number of mice. Statistical significance was defined as *P* < 0.05. All statistical tests were conducted in Prism5 (GraphPad Software, San Diego, CA, USA). For two groupings, a *t* test was conducted with the appropriate *post hoc* test. For more than two groupings, a one‐way ANOVA was conducted. If the data passed normality criteria (D'Agostino's test of normality and Bartlett's test of equal variances), a parametric test was conducted with the appropriate *post hoc* test (Tukey). If the normality criteria were not met, a Kruskal–Wallis test with Dunn's multiple comparison test was conducted.

Time‐series analysis of electrophysiological and Ca^2+^ imaging data was conducted in MATLAB v6.1 (2000; The MathWorks, Natick, MA, USA). Light‐pulse‐triggered peaks in membrane potential >20 mV were detected and averaged. These peaks were also used to determine firing frequencies before and during opto‐activiation.

## Computational methods

Models of the electrical activity in human islets were constructed. All models were coded in the hoc environment and simulated in NEURON using CVODE and a 25 μs timestep (Carnevale & Hines, 2006). Videos of these simulations can be accessed via the online Supporting Information.

### Morphology of human islet models

Experimental data of the cellular architecture of six human islets from a previously published study were used to define the morphology of the models (fig. 8 and table 2 in Hoang *et al*. 2014). For each islet, the data provide the spatial (*x*, *y*, *z*) location of each individual α‐, β‐ and δ‐cell within the islet. We then placed the appropriate model of electrical activity (α, β or δ, as given below) at each such location, creating six human islet models (M1–M6). The data also gave information about which cells are in ‘contact’ with one another (Hoang *et al*. 2014), which we used to endow the model with mechanisms of cell‐to‐cell communication.

### α‐cell model

As our focus is on understanding the regulation of α‐cell activity and glucagon secretion, the α‐cell model we developed was the most detailed. It built upon previously published models of the electrical activity in α‐cells (Diderichsen & Gopel, 2006; Fridlyand & Philipson, 2012; Watts & Sherman, 2014; Pedersen *et al*. 2016; Watts *et al*. 2016).

The equation describing membrane potential in the α‐cell model is:

(1) C cell dVdt=−(I CaL +I CaN +I CaT +I Na +IK+I KATP +I KA +IL+I GIRK )

where *C*_cell_ is the cell capacitance; *I*_CaL_, *I*_CaN_ and *I*_CaT_ are L‐, N‐ and T‐type voltage‐dependent Ca^2+^ currents, respectively; *I*_Na_ is a voltage‐dependent Na^+^ current; *I*_K_ is a delayed rectifier K^+^ current; *I*_KA_ is an A‐type voltage‐dependent K^+^ current; *I*_K(ATP)_ is an ATP‐sensitive K^+^ current; and $I_{L}$ is a leak current. Both human and mouse α‐cells express SST receptors that are coupled to G‐protein inwardly rectifying potassium (GIRK) channels (Braun, 2014). We therefore modified the recent model of Briant *et al*. (2017) to include the GIRK current, *I*_GIRK_. This had a maximal conductance density modulated by the local SST concentration, [SST] – a concentration determined by SST secretion from contacting δ‐cells.

We also modelled glucagon secretion. The intracellular calcium concentration, [Ca^2 +^ ], was modelled as the sum of calcium fluxes due to the total calcium current ( I Ca =I CaL +I CaN +I CaT ) and a calcium buffering term:

(2) d[Ca2+]dt=2I Ca Fd·d+([Ca2+]0−[Ca2+])τ

Here, calcium is buffered to [Ca^2 +^ ]_0_ with time‐constant τ, $F_{d}$ is Faradays constant and *d* is the depth of the calcium domain. This calcium concentration drives a system of differential equations describing glucagon vesicle dynamics:

(3) d[FA]dt=kb([F max ]−[FA]−[VA])[Ca2+]4−ku[FA]−k1[FA][V]+k2[VA]

(4) $$\frac{d[V_{A}]}{\mathrm{dt}}=k_{1}\left[F_{A}\right]\left[V\right]-\left(k_{2}+k_{3}\right)\left[V_{A}\right]$$

(5) $$\frac{d[\mathrm{Glg}]}{\mathrm{dt}}={\mathrm{Nk}}_{3}\left[V_{A}\right]-k_{h}\left[\mathrm{Glg}\right]$$

Here, calcium ions are assumed to reversibly bind to a fusion protein *F*. Four calcium ions bind to this protein at a rate *k*_b_, activating it. The concentration of activiated fusion protein is [*F*_A_], coming from a pool of inactivated proteins with concentration [*F*_max_]. The reverse process has an unbinding rate *k*_u_. An activated fusion protein binds to a vesicle (*V*) at a rate *k*_1_, activated it (*V*_A_). This process is reversible with unbind rate *k*_2_. The concentrations of inactivated and activated vesicles are [*V*] and [*V*_A_], respectively. Destexhe *et al*. (1994) simplified this system by assuming that there exists an inexhaustible pool of inactivated vesicles, ready for activation. In particular, [*V*] is constant and not depleted. This assumption is adopted. An activated vesicle is then able to fuse to the membrane of the cell, and release its contents. An activated vesicle releases *N* molecules of glucagon (Glg) at a rate *k*_3_. The concentration of glucagon released is [Glg]. This is depleted in the extracellular space by diffusion, degradation and reuptake at a rate *k*_h_.

### β‐cell model

There are many excellent models of the electrical activity in β‐cells that could be selected (reviewed by Pedersen, 2009). As we are interested in the regulation of α‐cell physiology, we chose the five variable model of Bertram *et al*. (2004). This simple model produces the desired bursting behaviour, which is observed in human β‐cells (Riz *et al*. 2014), whilst minimizing computational complexity.

### δ‐cell model

The equation describing membrane potential in the δ‐cell model was:

(6) C cell dVdt=−(I CaL +I CaN +I Na +IK+I KATP +I KA +IL)

where *C*_cell_ is the cell capacitance; *I*_CaL_ and *I*_CaN_ are the L‐ and N‐type voltage‐dependent Ca^2+^ currents, respectively; *I*_Na_ is a voltage‐dependent Na^+^ current; *I*_K_ is a delayed rectifier K^+^ current; *I*_KA_ is an A‐type voltage‐dependent K^+^ current; *I*_K(ATP)_ is an ATP‐sensitive K^+^ current; and$\;I_{L}$ is a leak current. This model is identical to the recent model of Briant *et al*. (2017), but we also included eqns (2)–(5) to model SST secretion.

### Modelling communication between electrically coupled cells

We have outlined the architectures and individual cellular components of the islet models. What remains to be described is how contacting cells communicate with one another.

#### GJ coupling between pairs of contacting β‐cells

All contacting β‐cells were considered to form a functional GJ with a strength (in pS) picked from a normal distribution of mean 40 pS and standard deviation 1 pS. Each cell may form 1–5 GJ connections with other cells, yielding a total mean GJ conductance of 40–200 pS. This is supported both by experimental and by simulation data from small clusters of dispersed human β‐cells (100–200 pS; Loppini *et al*. (2015)) and β‐cells recorded in intact mouse islets (<170 pS, Zhang *et al*. (2008); 50–120 unitary strength, Moreno *et al*. (2005); 20 pS, Perez‐Armendariz *et al*. (1991)).

#### GJ coupling between contacting δ‐ and β‐cells

β‐cells were also considered to be GJ coupled to δ‐cells (see Results). The strength of this coupling was treated as an unknown parameter. We constrained our considered range for this parameter to 0–100 pS, to mimic derivations of the coupling strength between small clusters of dispersed human β‐cells (Loppini *et al*. 2015).

#### Paracrine signalling from δ‐ to α‐cells

GIRK channels have been shown to underlie the hyperpolarizing K^+^ currents activated by SST in rodent (Yoshimoto *et al*. 1999) and human (Kailey *et al*. 2012) α‐cells. Instead of explicitly modelling SST receptor dynamics, we assumed that the released SST directly modulates the GIRK channel conductance (g GIRK ¯) in contacting α‐cells according to the equation:

(7) g GIRK ¯=k[SST]

where *k* has units of μS/mm.

### Cell‐to‐cell variability and parameter uncertainty

As shown by Briant *et al*. (2017), ionic and cellular electrophysiological properties can be very variable in pancreatic cells. To account for this variability and uncertainty in conductance values, within an islet model, parameter values for each individual cell were picked from a normal distribution (Table 1). This produces an islet model with cell‐to‐cell variability (within‐islet variability). This islet model was then simulated under conditions of low and high glucose. This process was repeated 100 times for each islet, yielding between‐simulation variability. This allowed us to account for variability and parameter uncertainty in our simulation results – as has been done in cardiac and neuronal modelling (Marder & Taylor, 2011; Sarkar *et al*. 2012; Walmsley *et al*. 2013; Muszkiewicz *et al*. 2016). All simulation results are expressed as means ± SEM of these 100 simulations.

**Table 1:** Model parameters picked from normal distributions with mean μ and standard deviation σ to account for parameter uncertainty

α‐cell model				β‐cell model				δ‐cell model			
Parameter	μ	σ	Units	Parameter	μ	σ	Units	Parameter	μ	σ	Units
*C*_cell_	4.2	0.1	pF	*C*_cell_	6.3	0.1	pF	*C*_cell_	4	0.1	pF
g Na ¯	0.11	0.01	S cm^−2^	g CaL ¯	0.003	0.01	S cm^−2^	g Na ¯	0.11	0.01	S cm^−2^
g KDR ¯	0.1	0.01	S cm^−2^	g KDR ¯	0.004	0.01	S cm^−2^	g KDR ¯	0.045	0.01	S cm^−2^
g CaL ¯	0.007	0.01	S cm^−2^	g Kslow ¯	0.003	0.01	S cm^−2^	g KA ¯	0.012	0.01	S cm^−2^
g CaN ¯	0.006	0.01	S cm^−2^	$\bar{g_{K,\;\mathrm{ATP}}}$	0.00048		S cm^−2^	g CaL ¯	0.0065	0.01	S cm^−2^
g CaT ¯	0.004	0.01	S cm^−2^	$GJ_{\beta \beta }$	40	1	pS	g CaN ¯	0.003	0.01	S cm^−2^
g pas ¯	0.001	0.01	S cm^−2^					g CaT ¯	0.005	0.01	S cm^−2^
$\bar{g_{K,\;\mathrm{ATP}}}$	0.0008		S cm^−2^					g pas ¯	0.0002	0.01	S cm^−2^
								$GJ_{\beta \delta }$	0–100	1	pS

The standard deviation in maximal conductance densities was chosen as 0.01 S cm^−2^ to mimic maximal conductance densities seen in neurons (Seutin & Engel, 2010). The mean gap junction conductance between β‐ and δ‐cells $\left(GJ_{\beta -\delta }\right)$ is an unknown parameter. The influence this parameter has on model output is considered in Fig. 8.

## Results

### Optogenetic activation of β‐cells triggers a strong inhibition of α‐cell electrical activity

**Figure 1:**
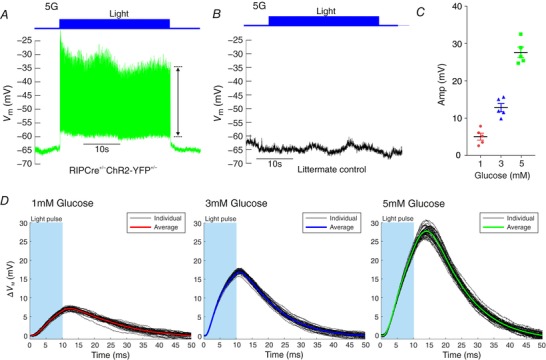
Opto‐activation of pancreatic β‐cells Electrical activity in β‐cells (*n* = 5) was recorded from islets under the perforated patch‐clamp configuration from RIPCre^+/−^ChR2‐YFP^+/−^ mice (*N* = 5). *A* and *B*, opto‐activation of ChR2 with 10 ms pulses of 488 nm light (20 Hz) triggered action potentials in β‐cells (*A*), but not in littermate controls (*B*). *C*, amplitude of the 20 Hz rhythm produced depended on the glucose concentration applied. In 5 mm glucose it was on average 27.6 ± 1.4 mV. *D*, example light‐driven membrane potential changes ($\Delta V_{m}$) in a β‐cell, in different glucose concentrations. Both individual and average sweeps are shown. [Color figure can be viewed at wileyonlinelibrary.com]

β‐cells were perforated patch‐clamped in islets isolated from RIPCre^+/−^ChR2‐YFP^+/−^ mice (*n* = 5) and optically excited with 20 Hz light pulses (Fig. 1). This generated electrical activity with a 20 Hz rhythm. The amplitude of this activity was 28 ± 1 mV (*n* = 5 cells, Fig. 1 *A* and *C*). The amplitude of this activity was glucose‐dependent, with only small amplitude (<15 mV) activity being possible in lower glucose concentrations (Fig. 1 *C* and *D*). In β‐cells from littermate controls, light stimulation did not evoke a change in membrane potential (Fig. 1 *B*).

**Figure 2:**
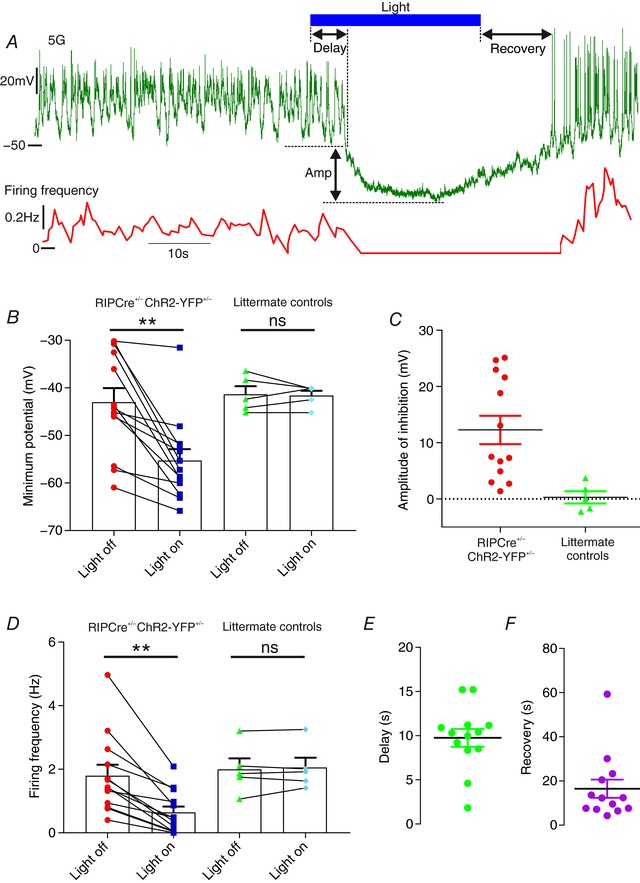
Opto‐activation of β‐cells triggers a strong suppression of α‐cell activity *A*, opto‐activation (20 Hz) of β‐cells caused a strong suppression of α‐cell (*n* = 13) electrical activity. The magnitude of this suppression was quantified by measuring the reduction in the minimal potential (*B*) and the amplitude of this reduction (*C*). *D*, firing frequency was also suppressed. The variance in firing frequency and minimum potential in the light‐off condition were no different in RIPCre^+/−^ChR2‐YFP^+/−^ compared to control α‐cells (Brown–Forsythe test; *P* = 0.10 and *P* = 0.60, respectively). The delay to suppression (*E*) and recovery to firing (*F*) were also characterized. Recordings from RIPCre^+/−^ChR2‐YFP^+/−^ mice (*N* = 7). Paired *t* test (^**^ *P* < 0.01). [Color figure can be viewed at wileyonlinelibrary.com]

Electrical activity in α‐cells from RIPCre^+/−^ChR2‐YFP^+/−^ mice exhibited strong inhibition in response to opto‐activation of β‐cells (Fig. 2). This was associated with a 12 mV hyperpolarization (Fig. 2 *B* and *C*) and a 65% reduction of the action potential frequency (Fig. 2 *D*). Interestingly, there was a ∼10 s delay between optogenetic activation of the β‐cell and α‐cell hyperpolarization (Fig. 2 *E*). Following cessation of optogenetic activation, the membrane potential returned to baseline values within 15–20 s (Fig. 2 *F*). Light stimulation had no effect on α‐cell electrical activity in islets from littermate controls (Fig. 2 *B* and *D*).

### Stimulation of β‐cells generates firing in δ‐cells

**Figure 3:**
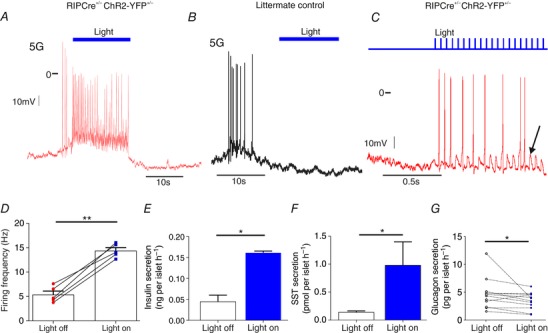
Opto‐activation of β‐cells triggers action potential firing in δ‐cells Opto‐activation (20 Hz) of β‐cells stimulated action potential firing in δ‐cells from RIPCre^+/−^ChR2‐YFP^+/−^ mice (*A*), but not littermate controls (*B*). The response was time‐locked to the light pulses (*C*). Subthreshold oscillations in membrane potential were also observed (arrow). The firing frequency of δ‐cells was increased by opto‐activation (*D*). *n* = 5 cells from 4 mice. Secretion of insulin (*E*) and SST (*F*) from RIPCre^+/−^ChR2‐YFP^+/−^ islets was increased in response to light stimulation (4 mice, 7 replicates). *G*, glucagon secretion from RIPCre^+/−^ChR2‐YFP^+/−^ islets was suppressed by 26% in response to light stimulation (4 mice, 12 replicates). Paired *t* test (^*^ *P* < 0.05; ^**^ *P* < 0.01). [Color figure can be viewed at wileyonlinelibrary.com]

In contrast to α‐cells, opto‐activation of β‐cells in RIPCre^+/−^ChR2‐YFP^+/−^ mice promptly stimulated δ‐cell electrical activity (Fig. 3 *A* and *D*). In littermate controls, light stimulation did not change the firing frequency of δ‐cells (Fig. 3 *B*). SST secretion in response to 20 Hz light pulses was also significantly increased (Fig. 3 *F*). Consistent with the observed hyperpolarization of α‐cells (Fig. 2), glucagon secretion was inhibited by 26% by opto‐activation of β‐cells (Fig. 3 *G*; *P* = 0.016), illustrating the efficient control of glucagon secretion by paracrine factors.

**Figure 4:**
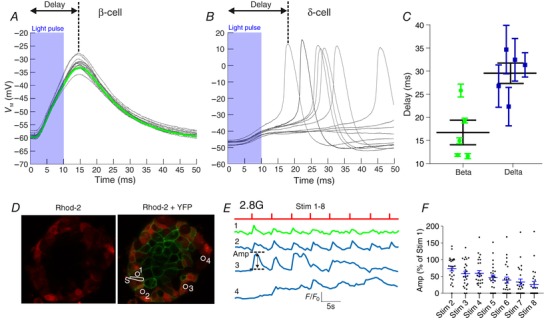
Excitation of δ‐cells by β‐cell opto‐activation has a delay consistent with GJ communication The time course of opto‐activation in (*A*) β‐cells (*n* = 5) was compared to (*B*) δ‐cells (*n* = 5). *C*, the difference in the delay to peak was 13 ms, similar to the calculated time taken for islet cells to charge one another via GJs (Zhang *et al*. 2008). Paired *t* test (^**^ *P* < 0.01). *N* = 5 mice. *D*, the time‐course of the Ca^2+^ response in δ‐cells to optogenetic stimulation of the membrane of a single YFP^+^ β‐cell was also investigated. The membrane of a single YFP^+^ β‐cell (1) was selected for photo‐stimulation (S). Cells loaded with Rhod‐2 (2–4) were then imaged for calcium responses. These cells were putatively δ‐cells because they *(i)* did not express YFP and *(ii)* were inactive in 2.8 mm glucose. *E*, example stimulus (S), response in the stimulated β‐cell (1) and four δ‐cells (2–4). *F*, amplitude of the Ca^2+^ signal in δ‐cells in response to repeated stimulation in *n* = 8 islets (*N* = 8 mice). [Color figure can be viewed at wileyonlinelibrary.com]

We next studied the time course of activation of δ‐ and β‐cells following opto‐activation (Fig. 4). The delay to peak potential following a 10 ms light pulse in β‐cells was only ∼17 ms (Fig. 4 *A* and *C*). Importantly, this delay was only marginally longer in δ‐cells (∼30 ms; Fig. 4 *B* and *C*), in contrast to the 10 s delay in α‐cells (Fig. 2 *E*). The difference in the delays between the initiation of electrical activity in β‐ and δ‐cells, following optogenetic activation of β‐cells (≈13 ms), suggests that δ‐cells are quickly excited following β‐cell activation. This delay difference is similar to calculations of the time needed for an islet cell to be charged via GJ connections with a neighbouring cell (Zhang *et al*. 2008). The time course of the Ca^2+^ response in δ‐cells was also investigated (Fig. 4 *D*–*F*). Spatially precise opening of ChR2 channels in the membrane of a single YFP^+^ β‐cell with the confocal laser generated a time‐locked Ca^2+^ response in δ‐cells (Fig. 4 *E*). We therefore postulated that the observed stimulation of membrane potential and intracellular Ca^2+^ in δ‐cells by opto‐activating β‐cells was via GJ coupling to β‐cells.

### δ‐cells are GJ coupled to β‐cells

**Figure 5:**
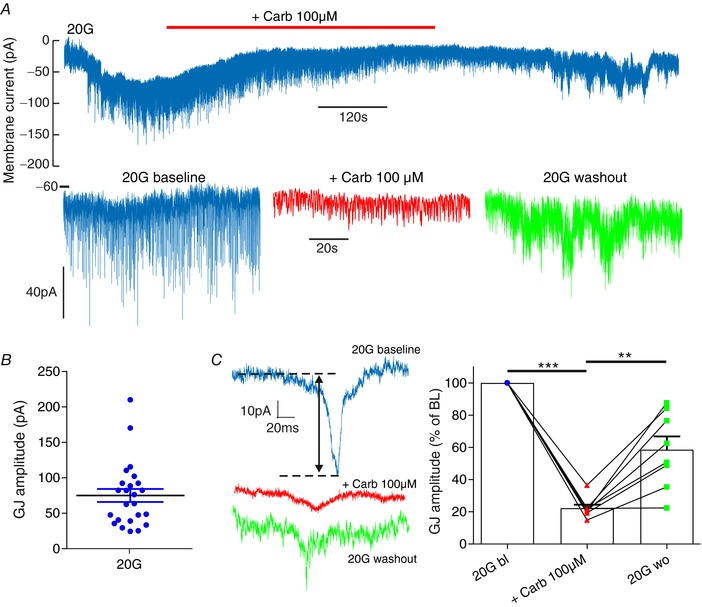
δ‐cells exhibit GJ currents δ‐Cells were patch‐clamped under the standard whole‐cell configuration from SST‐RFP mice (*N* = 6 mice). *A*, in high glucose (20 mm), when β‐cells will be electrically active, GJ currents were observed. *B*, the amplitude of these currents was quantified. *C*, application of the GJ inhibitor carbenoxolone (CARB, 100 μm; Juszczak & Swiergiel, 2009) caused a suppression of these currents (represented as % of baseline) that was partially reversed following washout. bl = baseline, wo = washout. One‐way repeated measures ANOVA (^**^ *P* < 0.01; ^***^ *P* < 0.001); *n* = 24 cell recordings. [Color figure can be viewed at wileyonlinelibrary.com]

To investigate the existence of GJ currents in δ‐cells, δ‐cells were patch‐clamped in SST‐RFP mice (Fig. 5). When islets were exposed to 20 mm glucose – a glucose concentration known to evoke electrical activity in mouse β‐cells (Antunes *et al*. 2000) – voltage‐clamped δ‐cells exhibited spontaneous inward current transients (Fig. 5 *A* and *C*). These currents represent action potentials fired in the neighbouring β‐cells that spread to the δ‐cell via GJs. These current transients had an amplitude of 75.1 ± 9.3 pA (*n* = 24 cells, Fig. 5 *B*). These GJ currents, expressed as a percentage of the amplitude at baseline, were abolished by application of CARB (22 ± 2.2%, *P* < 0.0001, *n* = 8 cells) and recovered following CARB washout (59 ± 8.3%, *P* = 0.004; Fig. 5 *C*). Taken together, these data support the presence of functional GJ connections between β‐ and δ‐cells. Given that β‐cell action potentials have an amplitude of 50 mV, the amplitude of the GJ current transients recorded in δ‐cells (75 pA) suggests that β‐ and δ‐cells are connected via a GJ conductance of 1.5 nS. This is similar to the 1.22 nS estimated for β‐cells (Zhang *et al*. 2008).

### α‐cell inhibition from β‐cells via δ‐cells

**Figure 6:**
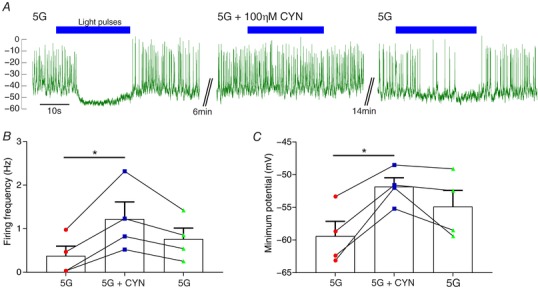
Inhibition of α‐cells by opto‐activation of β‐cells is mediated by δ‐cells *A*, the inhibition of α‐cell activity by opto‐activation of β‐cells is suppressed by application of the SSTR2 antagonist CYN 154806 (100 nm). CYN prevents the inhibition of (*B*) firing frequency and (*C*) membrane potential in α‐cells (*n* = 4) by opto‐activation. Recordings from RIPCre^+/−^ChR2‐YFP^+/−^ mice (*N* = 4). One‐way repeated measures ANOVA (^*^ *P* < 0.05). [Color figure can be viewed at wileyonlinelibrary.com]

The long delay between optogenetic activation of the β‐cell and membrane repolarization in the α‐cell (Fig. 2 *E*), and the slow reversal of this effect (Fig. 2 *F*), suggests the involvement of a diffusible factor. We hypothesized that the inhibition of α‐cell activity following opto‐activation of β‐cells in RIPCre^+/−^ChR2‐YFP^+/−^ mice (Fig. 2) was via GJ‐dependent activation of δ‐cells and subsequent stimulation of SST secretion (Figs 4 and 5), which inhibits glucagon secretion by activation of α‐cell somatostatin‐2 receptors (SSTR2). In support of this hypothesis, application of the SSTR2 inhibitor CYN 154806 during opto‐activation of β‐cells blocked the inhibition of electrical activity in α‐cells (Fig. 6 *A*–*C*).

Interestingly, the firing frequency in the presence of CYN 154806 was >100% higher than under control conditions (Fig. 6 *B*). Application of CYN 154806 was also associated with an 8 mV membrane depolarization (Fig. 6 *C*). These two effects suggest that SST is present under basal conditions to affect both membrane potential and α‐cell electrical activity.

### Simulation of human islets

To investigate whether the processes we have described above modulate α‐cell activity and glucagon secretion in human islets, we simulated our six human islet models (M1–M6) and modulated the degree of GJ coupling between β‐ and δ‐cells.

**Figure 7:**
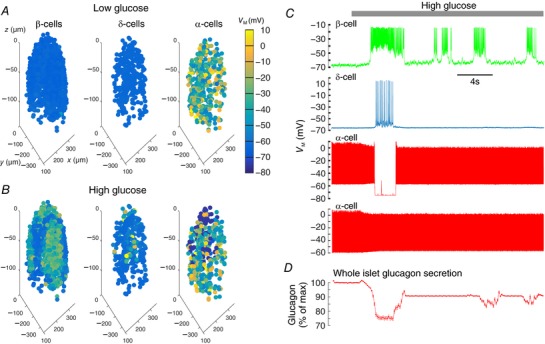
Simulation of human islets Architectures of six human islets were used to develop six models of electrical activity in human islets (see also Supplementary Videos S1–6). These architectures are from a previously published study (Hoang *et al*. 2014); the donor was a 51‐year‐old female with body mass index 29.3. *A*, islet Model 2 (M2) architecture and electrical activity in low glucose at *t* = 1 s. *B*, M2 architecture and electrical activity in high glucose at *t* = 5 s. *C*, time‐series traces of a selection of cells from M2 in response to high glucose. High glucose triggered bursting in β‐cells. This triggered firing in neighbouring δ‐cells via GJ connections. A neighbouring α‐cell exhibited suppressed firing, following SST release and GIRK channel activation. In comparison, distal α‐cells did not exhibit SST‐mediated suppression, but did exhibit a reduction in action potential height, as has been demonstrated experimentally (Zhang *et al*. 2013). *D*, the exocytosis of glucagon from each α‐cell was also modelled, and so could be quantified for the entire islet. [Color figure can be viewed at wileyonlinelibrary.com]

We first characterized the response of each islet model. Videos of simulations of M1–M6 can be accessed in the online supplementary material (Supporting Information, Videos S1–6). Typical time‐series for a selection of cells in model M2 are depicted in Fig. 7. This model has 430 α‐, 1468 β‐ and 366 δ‐cells. In low glucose, only the α‐cells were electrically active (Fig. 7 *A* and *C*). Application of high glucose generated bursting in β‐cells, which triggered action potential firing in a neighbouring δ‐cell via the GJs and evoked somatostatin secretion (Fig. 7 *B* and *C*). This in turn caused the suppression of firing in a neighbouring α‐cell (Fig. 7 *B* and *C*). Firing in an α‐cell not in contact with any δ‐cells was not suppressed by this paracrine signalling, but solely by intrinsic mechanisms (Fig. 7 *C*). Glucagon secretion from the whole islet could then be calculated (Fig. 7 *D*).

**Figure 8:**
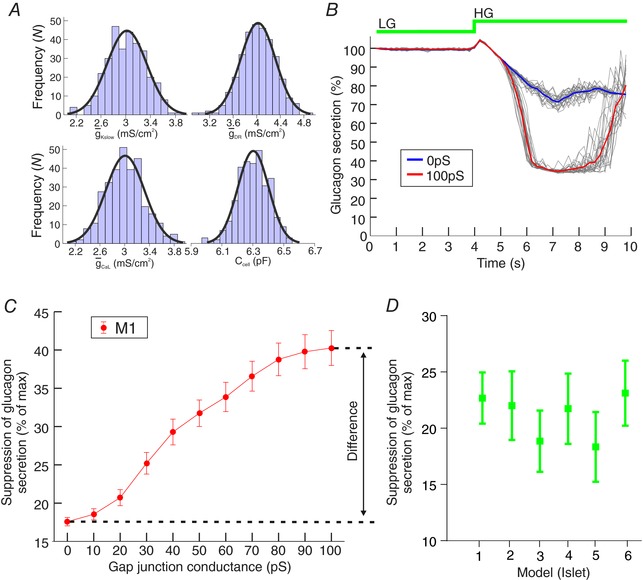
β‐to‐δ‐cell GJs regulate α‐cell activity and glucagon secretion in human islet simulations *A*, within an islet, parameter values were picked from normal distributions (Table 1) to account for cell‐to‐cell variability and parameter uncertainty. The model was then simulated under conditions of low and high glucose. This process was repeated 100 times to account for parameter uncertainty. *B*, the GJ conductance between β‐ and δ‐cells (GJ_β‐δ_) was fixed between 0 and 100 pS and the model simulated under low and high glucose conditions. The simulated glucagon secretion was then quantified and expressed as a % of that in low glucose. Grey lines = repeat simulations for re‐picked parameter values. *C*, suppression of glucagon secretion by high glucose (% of maximum) at different values of GJ_β‐δ_ in islet model M1. *D*, suppression of glucagon secretion for GJ_β‐δ_ = 0 pS and 100 pS in all six islet models (M1–M6). [Color figure can be viewed at wileyonlinelibrary.com]

We next explored the influence of β‐to‐δ‐cell GJ coupling on α‐cell activity and glucagon secretion in islet models M1–M6 (Fig. 8). For each model, we fixed the mean GJ conductance between β‐ and δ‐cells (GJ_β‐δ_) to be 0–100 pS (10 pS increments). As GJ_β‐δ_ was increased, the suppression of glucagon secretion by glucose increased in a sigmoidal fashion in all islet models. In model M1, when GJ_β‐δ_ = 100 pS, the suppression of glucagon secretion by high glucose was 40.4%, compared to 17.3% when GJ_β‐δ_ = 0 pS (Fig. 8 *C*). The difference, 23.1%, gives the contribution of β‐to‐δ‐cell GJ to this suppression. This contribution differed slightly across the islet models but was on average 21 ± 1% (*n* = 6, Fig. 8 *D*).

## Discussion

**Figure 9:**
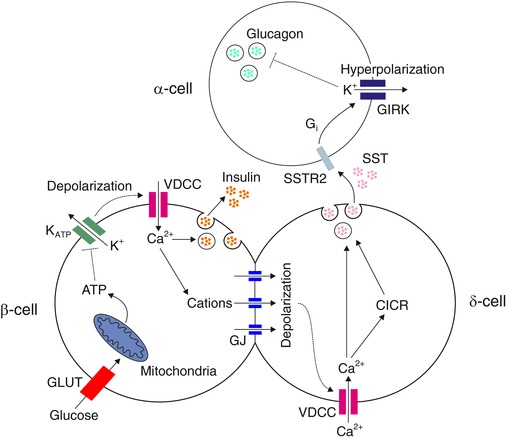
Schematic summary of the β‐to‐δ GJ pathway regulating α‐cell activity In conditions of high glucose, glucose is transported into β‐cells via glucose transporters (GLUT) and metabolized by the mitochondria. The consequential increase in ATP concentration closes K_ATP_ channels, causing membrane depolarization, Ca^2+^ entry (via voltage‐dependent Ca^2+^ channels; VDCCs) and therefore insulin secretion. This secreted insulin may directly regulate glucagon secretion (Kawamori *et al*. 2009), but we show that the aforementioned depolarization also drives cation flow through gap junctions (GJ) with coupled δ‐cell(s). This leads to depolarization of the δ‐cell, triggering Ca^2+^ entry via VDCCs. This entry, together with Ca^2+^‐induced Ca^2+^ release (CICR; Zhang *et al*. 2007) from intracellular Ca^2+^ stores, drives somatostatin (SST) secretion. Glucose may also directly activate δ‐cells by a K_ATP_‐dependent mechanism (not depicted; Braun *et al*. 2009; Gopel *et al*. 2000). Released SST binds to SST receptor 2 (SSTR2) on α‐cells, triggering a G‐protein cascade (G_i_) that activates G‐protein coupled inwardly rectifying K^+^ (GIRK) channels. This hyperpolarizes the α‐cell, leading to suppression of glucagon secretion. [Color figure can be viewed at wileyonlinelibrary.com]

In this study we used an optogenetic strategy to investigate the paracrine regulation of α‐cells. We used a mouse model that expressed ChR2 specifically in β‐cells (Reinbothe *et al*. 2014). Using the perforated patch‐clamp technique, we could investigate the electrophysiological consequences of opto‐activating β‐cells on α‐ and δ‐cells. We found that activating β‐cells results in a strong suppression of α‐cell activity (Fig. 2) and that this suppression is mediated via δ‐cells (Figs 3, 4 and 6). The excitation of δ‐cells was due to functional GJ connections between β‐ and δ‐cells (Figs 4 and 5). We therefore propose that α‐cell activity in mouse islets is regulated by β‐cells via GJ connections to δ‐cells (a schematic summary of this pathway is depicted in Fig. 9).

To investigate the contribution of this pathway in human islets, we constructed mathematical models of six human islets and carried out simulations under conditions of low and high GJ connectivity between β‐ and δ‐cells (Figs 7 and 8). The simulations demonstrated that in human islets the β‐to‐δ‐cell GJ pathway accounts for ∼23% of the suppression of glucagon secretion in high glucose (Fig. 8).

### Functional GJ connections between β‐ and δ‐cells

β‐cells are electrically coupled (Meissner, 1976; Perez‐Armendariz *et al*. 1991; Moreno *et al*. 2005; Zhang *et al*. 2008). In mouse (Moreno *et al*. 2005; Ravier *et al*. 2005) and human (Serre‐Beinier *et al*. 2009) this is mediated via the GJ protein connexin‐36 (Cx36). In islets, these junctions preferentially exchange cationic molecules (Charpantier *et al*. 2007), allowing charge to pass between β‐cells. This results in the entrainment of β‐cell activity and the recruitment of electrically silent β‐cells. Evidence has highlighted how crucial this coupling is for the appropriate secretion of insulin in response to changing glucose levels; deletion of Cx36 results in aberrant insulin secretion and a loss of cell‐to‐cell synchrony (Ravier *et al*. 2005; Speier *et al*. 2007). Clearly, GJs have a crucial role in the regulation of insulin output from islets.

In contrast, the role of GJs in the regulation of glucagon and SST secretion from α‐ and δ‐cells, respectively, remains unknown. Studies have detected GJ‐forming proteins in non‐β‐cell fractions in mouse (Meda, 2013) and human islets (Serre‐Beinier *et al*. 2009), with single cell RNA sequencing recently reporting the presence of GJ transcripts in δ‐cells (DiGruccio *et al*. 2016). Despite these observations, functional GJs have only been recorded between β‐cells [see reviews by Farnsworth and Benninger (2014) and Meda (2013)]. In this study, we provide evidence that δ‐cells are GJ connected to β‐cells.

Our demonstration of GJ coupling between β‐ and δ‐cells is based on several observations. The first is that δ‐cells exhibited CARB‐sensitive membrane currents in the standard whole‐cell voltage‐clamp configuration (Fig. 5). We note that the concentration of CARB used (100 μm) is known to block electrical coupling between central neurones, but has also been shown to have off‐target effects (see review by Connors 2012). However, this does not undermine our additional evidence for GJ coupling between β‐ and δ‐cells; as well as recording GJ currents in δ‐cells, we demonstrate that δ‐cell action potentials can be triggered by opto‐activation of β‐cells (Fig. 3) with a temporal delay similar to that observed for GJ‐coupled β‐cells (Fig. 4; Zhang *et al*. 2008). Furthermore, we also show that opto‐activation of β‐cells causes a stimulation of SST secretion (Fig. 3). Together, these data demonstrate the existence of functional GJ connections between β‐ and δ‐cells. This pathway explains the coincidence in pulses of insulin and SST secretion generated in response to glucose application in human (Hellman *et al*. 2009) and mouse (Salehi *et al*. 2007) islets.

### Which proteins form GJs between β‐ and δ‐cells?

Recently, single‐cell RNA sequencing data have demonstrated the presence of transcripts for pannexin (*Panx1* and *Panx2*) and connexin (*GJA4*) proteins in δ‐cells (DiGruccio *et al*. 2016). In our study we observed GJ currents in δ‐cells that were blocked by CARB. This drug is both a general connexin blocker (Giaume & Theis, 2010) and an inhibitor of cell‐to‐cell connections formed by pannexins (Michalski & Kawate, 2016). Our data also demonstrate that the β‐to‐δ‐cell GJ pathway leads to suppression of α‐cell activity. In keeping with our data is the recent observation that *Panx1*‐null transgenic mice have an increase in basal glucagon release (Cigliola *et al*. 2015). Together, these findings suggest that the β‐to‐δ cell GJ connectivity we observe may be formed by pannexins.

### β‐to‐δ GJ connections regulate α‐cell activity in mouse islets: a novel paracrine pathway

It is not surprising that stimulation of β‐cells generates an inhibitory response in α‐cells. The pathway we describe can be added to the list of paracrine signals that inhibit glucagon secretion [reviewed by Gromada *et al*. (2007), Caicedo (2013), Briant *et al*. (2016) and Gylfe (2013)], including insulin (Kawamori *et al*. 2009), Zn^2+^ (Ishihara *et al*. 2003), serotonin (Almaca *et al*. 2016), γ‐hydroxybutyrate and glycine (Li *et al*. 2013). Indeed, glucagon secretion was inhibited by 26% by opto‐activation of β‐cells over the hour‐long secretion protocol (Fig. 3 *G*); although this is consistent with our simulations, the suppression is probably due to a combination of multiple paracrine pathways, rather than solely due to the pathway we describe. What was surprising was that application of the SSTR2 inhibitor CYN 154806 completely abolished α‐cell hyperpolarization, due to β‐cell activation (Fig. 6). This cannot be explained by non‐specific expression of ChR2 in δ‐cells because fluorescence‐activated cell sorting has confirmed that ChR2 expression is highly restricted to β‐cells (only 0.5 ± 0.1% of YFP^+^ cells are non‐β‐cells) in this mouse model (Reinbothe *et al*. 2014). Acute exposure of CYN 154806 produces intrinsic activity when it is receptor‐bound (Nunn *et al*. 2003). To minimize the influence of this intrinsic activity in our patch‐clamp experiments, our application of CYN 154806 was kept brief (8–10 min). We did not test whether CYN 154806 blocks the β‐cell‐mediated inhibition of glucagon secretion.

In mouse islets, the favourability of δ‐to‐α communication due to their close proximity was first postulated by Orci & Unger (1975). In this study we demonstrate that this paracrine pathway between δ‐ and α‐cells is recruited when β‐cells become active, via GJ connections with δ‐cells. These findings do not preclude paracrine mechanisms observed in other studies; in fact, the studies of Almaca *et al*. (2016) and Li *et al*. (2013) were conducted in human islets – a paracrine environment very architecturally different from that of mouse islets (Brissova *et al*. 2005; Cabrera *et al*. 2006). For this reason, we also sought to assess whether this GJ pathway regulates α‐cell activity and glucagon secretion in human islets. Given the variability in human islet quality, function, donor details and availability (Ihm *et al*. 2006; Hanson *et al*. 2010; Kayton *et al*. 2015), we opted for the construction of mathematical models of human islets in order to make this assessment.

### Simulations of human islets

We developed six models of human islets. To our knowledge, no such multicellular and architecturally detailed models of islet electrical activity have been constructed. We ensured our models accounted for parameter uncertainty and cell‐to‐cell variability, as has been demonstrated in large datasets of electrophysiological recordings of islet cells (Briant *et al*. 2017).

Our simulations were able to capture some fundamental aspects of the electrophysiological response to glucose, including suppression of glucagon secretion by high glucose (Fig. 7). Furthermore, when we increased the degree of coupling between β‐ and δ‐cells to 100 pS, we saw a 23% increase in the suppression of glucagon secretion by high glucose (Fig. 8). Our mathematical models of islets therefore suggest that 23% of the inhibition of glucagon secretion by high glucose can be attributed to the β‐to‐δ GJ pathway, remarkably similar to the inhibition in our secretion experiments (26%; Fig. 3 *G*).

These simulations have significance not only for the normal regulation of glucagon secretion, but also for T2DM. Islet architecture is altered in T2DM, with islets from T2DM donors exhibiting fewer β‐to‐δ contacts (Kilimnik *et al*. 2011). In the context of our finding of a β‐to‐δ GJ pathway, such a reduction in β‐to‐δ cell contacts may explain the exacerbation of glucagon secretion in high glucose characteristic of T2DM (Cryer, 2008). Furthermore, animal models with a hyperglycaemic phenotype have reported a reduction of the number of GJs between islet cells (Carvalho *et al*. 2012; Haefliger *et al*. 2013). This can be predicted to have two consequences: first, a decrease in insulin secretion; and second, a reduction in the suppression of glucagon secretion by the β‐to‐δ‐cell GJ pathway described here. This may contribute to the hyperglucagonaemia under hyperglycaemic conditions that is a characteristic of T2DM and that exacerbates the consequences of the insulinopaenia (Cryer, 2008). It remains to be seen whether these findings in T2DM are indeed extended to coupling between β‐ and δ‐cells.

### Study limitations and future directions

One of our implicit modelling assumptions was that immunocytochemically established contact between cells (Hoang *et al*. 2014) implies functional GJ connectivity between cells. Contact between cells does not imply GJ coupling, but information pertaining to the electrical connectivity of all cells in an islet would be extremely difficult to obtain. To account for this, we treated GJ connectivity between contacting cells as a free parameter; and explored how the model output depended on this parameter (Fig. 8) as well as allowing it to vary according to a normal distribution (Table 1). We also recognize that the immunocytochemical techniques used by Hoang *et al*. (2014) to characterize islet architecture may not have the level of precision required to capture all cell‐to‐cell contacts. For example, δ‐cells are known to have long projections that extend beyond their immediate neighbourhood; Brereton *et al*. (2015) demonstrated with sequential electron microscopy images that rat δ‐cells have projections that extend >50 μm, contacting multiple cells. These projections could not be captured by the methodology of Hoang *et al*. (2014). However, the extent and function (if any) of these projections have yet to be fully characterized in mouse or human islets, and so were not included in our models.

One may expect that we could correlate our simulation findings with measures of islet architecture. For example, does the degree of suppression of glucagon secretion by high glucose correlate with the number of δ‐cells, the number of δ‐to‐α contacts, or graph theory measures of connectivity and centrality (Striegel *et al*. 2015)? The limiting factor in answering these questions is our sample size – we only have six human islet architectures. As a future direction, we aim to conduct simulations on a larger sample of human islet architectures, so that we may correlate islet architecture with islet function in islets from non‐diabetic and T2DM human donors.

## Additional information

### Competing interests

The authors declare no competing interests.

### Author contributions

L.J.B.B. conceived the study design, drafted the manuscript, recorded the experimental data (patch‐clamp), constructed and simulated the mathematical models, and analysed the experimental and computational data. C.M and T.M.R. managed the mouse colonies, recorded the experimental data (Ca^2+^ imaging), provided intellectual input and helped draft the manuscript. I.S. recorded the experimental data (secretion), provided intellectual input and helped draft the manuscript. P.R. and B.R. provided financial support for the project, significant intellectual input and helped draft the manuscript. All authors approved the final version of the manuscript.

### Funding

LJBB is funded by a Sir Henry Wellcome Postdoctoral Fellowship (Wellcome Trust, 201325/Z/16/Z). Financial support was also received from Wellcome Trust grant numbers 884655, 089795 and 095531. BR is funded by a Welcome Trust Senior Research Fellowship in Basic Biomedical Science (100246/Z/12/Z), the British Heart Foundation Centre of Research Excellence in Oxford (RE/13/1/30181), an NC3R Infrastructure for Impact award (NC/P001076/1), an EPSRC Impact Acceleration Award (EP/K503769/1) and the ComBioMed project funded by the European Commission (grant agreement No 675451)). Funding was also provided by the Swedish research council (VR), Wilhems and Martina Lundgrens Research Fund, Sigurd and Elsa Goljes Memory Foundation and Adlerbert Research Foundation.

## Supporting information

Disclaimer: Supporting information has been peer‐reviewed but not copyedited.

**Video S1**. Simulation of high glucose in human islet model M1. Simulation of model of first islet architecture in high glucose. This islet has 150 α‐, 319 β‐ and 122 δ‐cells.

**Video S2**. Simulation of high glucose in human islet model M2. Simulation of model of second islet architecture in high glucose. This islet has 430 α‐, 1468 β‐ and 366 δ‐cells.

**Video S3**. Simulation of high glucose in human Islet model M3. Simulation of model of third islet architecture in high glucose. This islet has 1093 α‐, 1544 β‐ and 619 δ‐cells.

**Video S4**. Simulation of high glucose in human islet model M4. Simulation of model of fourth islet architecture in high glucose. This islet has 970 α‐, 2256 β‐ and 351 δ‐cells.

**Video S5**. Simulation of high glucose in human islet model M5. Simulation of model of fifth islet architecture in high glucose. This islet has 650 α‐, 1174 β‐ and 275 δ‐cells.

**Video S6**. Simulation of high glucose in human islet model M6. Simulation of model of sixth islet architecture in high glucose. This islet has 838 α‐, 1362 β‐ and 661 δ‐cells.
